# Tamoxifen Blocks the Development of Motivational Features of an Addiction-Like Phenotype in Female Rats

**DOI:** 10.3389/fnbeh.2019.00253

**Published:** 2019-11-08

**Authors:** Anousheh Bakhti-Suroosh, Tanseli Nesil, Wendy J. Lynch

**Affiliations:** Department of Psychiatry and Neurobehavioral Sciences, University of Virginia, Charlottesville, VA, United States

**Keywords:** cocaine, estradiol, extended access, self-administration, sex differences

## Abstract

Women become addicted sooner after initiating cocaine use as compared to men. Preclinical studies reveal a similar vulnerability in females, with findings from ovariectomized rats suggesting that estradiol mediates the enhanced vulnerability. However, since ovariectomy depletes not only estradiol, but all ovarian hormones, its role in a physiological context is not clear. Thus, the goal of this study was to determine the role of estradiol in the development of an addiction-like phenotype in ovary-intact females treated chronically with the selective estrogen receptor (ER) modulator tamoxifen. We hypothesized that tamoxifen, by antagonizing ERs, would block the development of an addiction-like phenotype as defined by an enhanced motivation for cocaine (assessed under a progressive-ratio schedule), and a heightened vulnerability to relapse (assessed under an extinction/cue-induced reinstatement procedure). Effects were examined following extended access cocaine self-administration (24-h/day; 4-discrete trials/h; 1.5 mg/kg/infusion) and 14-days of abstinence, conditions optimized for inducing an addiction-like phenotype. As predicted, motivation for cocaine was increased following extended-access self-administration and protracted abstinence in the vehicle (sesame oil) and no-injection control groups, but not in the tamoxifen group indicating that ER signaling is critical for the development of this feature of an addiction-like phenotype. Surprisingly, the increase in motivation for cocaine following abstinence was also attenuated in the vehicle group as compared to no-injection controls suggesting that oil/injections also affected its development. Contrary to our hypothesis, tamoxifen did not decrease vulnerability to relapse as this group responded at similar levels during initial extinction sessions and cue-induced reinstatement testing as compared to controls. Tamoxifen did, however, impair extinction learning as this group took longer to extinguish as compared to controls. Taken together, these findings indicate that estradiol is critical for the extinction of drug-associated cues and the development of motivational features of addiction.

## Introduction

Cocaine use is a leading cause of overdose deaths in the U.S., second only to opioids (Hedegaard et al., 2018). Among African-Americans, it’s the number one cause (Shiels et al., 2018), with rates of overdose deaths from cocaine on par with, or exceeding, overdose deaths from opioids in white Americans (Shiels et al., 2018). Furthermore, cocaine use is on the rise with 2.2 million Americans reporting current use in 2017 vs. 1.9 million in 2016 (Center for Behavioral Health Statistics and Quality, 2015; Substance Abuse and Mental Health Services Administration, 2017)—a trend predicted to continue as levels of coca cultivation and potential cocaine production in Colombia rise (United States Drug Enforcement Administration, 2016). Women users are particularly concerning as they become addicted to the drug more quickly and display more serious drug-related medical and psychological complications (Center for Substance Abuse Treatment, 2009; Greenfield et al., 2010; Becker and Koob, 2016). Once addicted, women also have longer periods of use after relapse, relapse for different reasons, and have a greater risk of admittance to treatment facilities as compared to men (White et al., 1996; Luchansky et al., 2000; Gallop et al., 2007; Potenza et al., 2012).

Preclinical studies also reveal a faster time-course for the development of features of an addiction-like phenotype in females vs. males (Lynch and Carroll, 2000; Lynch and Taylor, 2004; Kawa and Robinson, 2019; Nicolas et al., 2019). For example, female rats given extended access (ExA) to cocaine (6–24 h/day) take more cocaine and display a greater disruption of diurnal control over intake as compared to males (Lynch and Taylor, 2004, 2005; Roth and Carroll, 2004; Kawa and Robinson, 2019; Nicolas et al., 2019). Furthermore, females show an enhanced motivation for cocaine following 7 days of ExA self-administration and 10 days of abstinence—conditions that do not impact motivation for cocaine in males (Lynch and Taylor, 2004). However, when conditions are optimized (i.e., 10 days of ExA self-administration and 14 days of abstinence), both males and females show an enhanced motivation for cocaine (Ramôa et al., 2013). These findings are consistent with humans and indicate that the enhanced time-course for the development of cocaine addiction in females is biologically-based.

The biological mechanisms underlying these sex differences are unknown but likely involve the ovarian hormone estradiol (Segarra et al., 2010; Quinones-Jenab and Jenab, 2012; Ramôa et al., 2013). Women report the greatest sensitivity to the euphoric effects of cocaine and other stimulants during the follicular phase when estradiol levels are high and progesterone levels low (Justice and de Wit, 1999; Evans et al., 2002). Similarly, in female rats, motivation for cocaine and cocaine-seeking vary across the estrous cycle with the highest levels observed during estrus, when the ratio of estradiol to progesterone is relatively high (Lacy et al., 2019; Nicolas et al., 2019). Additionally, ovariectomy (OVX) has been reported to decrease cocaine self-administration under ExA conditions (Larson et al., 2007; Martinez et al., 2016), and to prevent the development of an enhanced motivation for cocaine, even when assessed under optimized conditions (Ramôa et al., 2013). Notably, estradiol replacement restores both levels of self-administration and the development of an enhanced motivation for cocaine (Ramôa et al., 2013), indicating that estradiol may be necessary for the development of an addiction-like phenotype in females.

One important caveat, however, is that OVX depletes all ovarian hormones, not just estradiol. OVX also results in the cessation of rhythmic fluctuations in levels of hormones, which may be critical for effects on addiction (Di Paolo, 1994; Bossé et al., 1997; Zhang et al., 2008; Segarra et al., 2014). Thus, the role of estradiol in a physiological context is not clear. This is especially important considering that findings obtained in ovary-intact females are somewhat contradictory to those observed in OVX females in that levels of drug intake, motivation, and seeking are highest during estrus, and not proestrus when levels of estradiol peak (Kippin et al., 2005; Feltenstein and See, 2007). Furthermore, the activity of dopaminergic neurons in the ventral tegmental area is lowest in intact females during proestrus and highest during estrus (Zhang et al., 2008). While the ratio of estradiol to progesterone has been suggested as a means to reconcile the differential results observed in intact vs. OVX rats, neurochemical data in OVX rats showing that both estradiol and progesterone similarly increase dopamine release in the nucleus accumbens (NAc) contradict this idea (Zhang et al., 2008). These disparities indicate a need to explore the role of estradiol in the development of an addiction-like phenotype in ovary-intact females.

Thus, in this study, we examined the effects of chronic treatment with tamoxifen, a selective estrogen receptor (ER) modulator, on the development of an addiction-like phenotype in ovary-intact females. Tamoxifen has been used extensively as an ER antagonist both clinically, primarily as a treatment for breast cancer (Huang et al., 2015), as well as preclinically, in intact and estradiol-treated OVX female rats and mice since it readily crosses the blood-brain barrier, inhibits estradiol-dependent behaviors (i.e., lordosis), and antagonizes both alpha and beta ERs (Halbreich and Kahn, 2000; Wilson et al., 2003; Smith and O’Malley, 2004; Flynn et al., 2017; Sá et al., 2018). Tamoxifen has also been reported to block estradiol-induced increases in striatal dopaminergic signaling in OVX females (Ferretti et al., 1988; McDermott et al., 1998, 1999; Dluzen et al., 2001; Landry et al., 2002), and to prevent the acquisition of cocaine self-administration (Lynch et al., 2001), the development of tolerance to opioids (Chiang et al., 2017; Withey et al., 2017), and the expression of a morphine-induced conditioned place preference in gonad-intact rats and mice (Esmaeili et al., 2009). It also attenuates the acquisition of an estradiol-induced conditioned place preference and estradiol-induced anxiolytic effects in OVX and ovary-intact females (Walf and Frye, 2005; Walf et al., 2007; Azizi-Malekabadi et al., 2015).

As in our previous work (Ramôa et al., 2013), the development of an addiction-like phenotype was assessed by comparing motivation for cocaine prior to and following ExA self-administration and 14 days of abstinence. We also examined the effect of tamoxifen on the development of another key feature of addiction, relapse vulnerability, as measured following protracted abstinence using an extinction/cue-induced reinstatement procedure. Tamoxifen’s effects were compared to effects observed in vehicle-treated rats. We also included additional groups of non-treated rats as a control for the effects of daily vehicle treatment (sesame oil). We hypothesized that chronic tamoxifen treatment in ovary-intact females would prevent the development of an addiction-like phenotype, including an enhanced motivation for the drug and heightened vulnerability to relapse.

## Materials and Methods

### Animals

Subjects were sexually mature intact female (*N* = 62) Sprague-Dawley rats (Charles River), weighing 235–300 g at the start of the study. Upon arrival, rats were individually housed in operant testing chambers (Med Associates, St. Albans, VT, USA) and randomly assigned to one of two groups: vehicle-treated (VEH, *n* = 24) or tamoxifen-treated (TAM, *n* = 20). We also included additional non-treated, no-injection (NO INJ, *n* = 18) controls since initial results indicated that the development of an enhanced motivation for cocaine was attenuated by VEH treatment/injections (Lynch and Taylor, 2004; Ramôa et al., 2013). This group was run contemporaneously with the TAM and VEH groups as a control for vehicle injections. Throughout the study, rats were maintained on a 12-h light/dark cycle (house and room lights on at 7.00 AM), with *ad libitum* access to food and water (except as noted below for some animals during cocaine self-administration training). Following a 2-day habituation period, in order to encourage rapid subsequent acquisition of cocaine self-administration, rats were pre-trained to lever press for sucrose pellets (45 mg) using methods previously described (fixed-ratio 1; 24-h/day sessions; ≥50 sucrose pellets/session for 2 days; Lynch, 2008). Body weights were recorded three times/week and health was examined daily. The University of Virginia Animal Care and Use Committee approved all animal protocols, which adhered to the guidelines set by the National Institute of Health.

### Tamoxifen Treatment and Vaginal Cytology

Rats were given subcutaneous injections of tamoxifen (1.0 mg/kg) or an equal volume of sesame oil (~0.3 ml) between 8:30 and 11:30 AM 5 days/week beginning 1 day after arrival and continuing throughout the duration of the study, with the dose adjusted three times/week based on changes in body weight. Based on findings showing that a 5-day treatment regimen with estradiol prevents changes in dopamine receptor sensitivity that occur following daily administration, tamoxifen and vehicle treatments were administered 5 days/week (Di Paolo et al., 1981). Rats in the NO INJ group were handled similarly but did not receive injections. In order to verify the effectiveness of the tamoxifen treatment and to determine estrous cycle phase, vaginal samples were collected daily during the first week of treatment, and thereafter, weekly. Swabs were examined under light microscopy and the phase of the estrous cycle was determined using methods previously described (Lynch et al., 2001; Lynch and Taylor, 2005). Vaginal smears obtained from rats in the TAM group contained predominantly necrotic epithelia and leukocytes, which are indicative of metestrus and diestrus.

### Surgery and Catheter Maintenance

Following lever pre-training, rats were anesthetized with ketamine/dexdomitor in order to implant a chronic, indwelling catheter (Silastic tubing; 0.51 and 0.94 mm o.d.; Dow Corning, Midland, MI, USA) into the right jugular vein, using methods previously described (Lynch, 2008). Catheter patency was tested 3 days/week by flushing with heparinized saline (~0.5 ml), and by periodically administering methohexital (1.5 mg/kg). During the 14-day abstinence period, rats were given daily infusions of cefazolin (17 mg/kg) to help maintain patency. If a catheter was leaking, pressure prevented flushing, or the animal did not lose muscle tone immediately following the infusion of methohexital, the catheter was considered no longer patent and data collected between this assessment and the last patency check were discarded. If patency was lost, a new catheter was implanted into the left jugular vein. Cocaine self-administration resumed after 1–2 days of recovery.

### Experimental Procedures

#### Cocaine Self-administration Training

Rats were initially trained to self-administer cocaine (1.5 mg/kg/infusion) under a fixed-ratio 1 schedule with a maximum of 20 infusions/day, using methods previously described (Lynch et al., 2010). Acquisition was defined as two consecutive days wherein all 20 infusions were obtained. A relatively high dose of cocaine was used to encourage rapid rates of acquisition and moderate food restriction (20 g/day) was used briefly (2–3 days) when necessary. All groups acquired rapidly under these high dose conditions and rates of acquisition did not differ between groups. Responses on the right (non-active) lever were counted during self-administration sessions as a measure of general activity, but they did not have any programmed consequence.

#### Motivation for Cocaine

Following acquisition, motivation for cocaine was assessed using a progressive-ratio (PR) schedule wherein the response requirement to obtain a cocaine infusion increased progressively throughout the session in the following steps: 1, 2, 4, 6, 9, 12, 15, 20, 25, 32, 40, 50, 62, 77, 95, 118, 145, 178, 219, 268, 328, 402, 492, 603, etc. PR sessions were conducted as previously described (Ramôa et al., 2013), and were run for 22-h each day (responding typically ceased within 2–4 h) until a stable baseline was achieved (defined as no increasing or decreasing trend in the number of infusions obtained over three consecutive sessions; typically achieved within 3–4 sessions). The moderate dose of cocaine tested (0.5 mg/kg/infusion) has been shown to reveal motivational differences between OVX females with and without estradiol replacement following ExA self-administration and abstinence, while producing comparable levels of responding at baseline (Ramôa et al., 2013).

#### ExA Cocaine Self-administration

After achieving a stable PR baseline, rats were given ExA (24-h/day) to cocaine under a discrete trial procedure using methods previously described (1.5 mg/kg/infusion, 4-discrete trials/h, 10 days; Ramôa et al., 2013, 2014). Briefly, 10-min trials began every 15 min (96 infusions/day) with the extension of the active-lever into the chamber; after either 10 min or a response on the active-lever the trial was terminated and the lever retracted. These conditions have been shown to induce high levels of cocaine intake and dysregulated patterns of use (Ramôa et al., 2013). After the last ExA session, responding was again assessed under a fixed-ratio 1 schedule with a maximum of 20 infusions in order to equate levels of cocaine intake between groups before abstinence. A 14-day abstinence period began following the second fixed-ratio 1 session, during which animals remained in their test chambers.

#### Enhanced Motivation for Cocaine

In order to determine the impact of tamoxifen on the development of an addiction-like phenotype, motivation for cocaine was assessed following ExA self-administration and abstinence. This was conducted in a subset of rats (VEH, *n* = 16; TAM, *n* = 13; NO INJ, *n* = 10) using a PR schedule as described above.

#### Enhanced Relapse Vulnerability

The impact of tamoxifen on vulnerability to relapse was assessed in a subset of rats (VEH, *n* = 8; TAM, *n* = 7; NO INJ, *n* = 8) following ExA cocaine self-administration and 14 days of abstinence using a within-session extinction/cue-induced reinstatement procedure and methods previously described (Lynch et al., 2010). Briefly, extinction responding was examined in a minimum of 6, 1-h extinction sessions, and once responding extinguished (<15 responses) or a maximum of nine extinction sessions, reinstatement responding elicited by the cues formerly associated with cocaine (light above the lever and sound of the pump) was assessed in a 1-h session.

### Hormone Measurements

Serum concentrations of estradiol and progesterone were assessed in a subset of rats using methods previously described (Lynch, 2008). Trunk blood collection occurred between 10 AM and 12 PM following the completion of the last PR session (VEH, *n* = 6; TAM, *n* = 7; NO INJ, *n* = 7) or the extinction/reinstatement test (VEH, *n* = 6; TAM, *n* = 4; NO INJ, *n* = 7). Rats in the PR experiment also underwent pharmacological testing prior to serum collection; however, this testing did not appear to impact hormone levels as no differences were observed for either estradiol or progesterone levels in serum collected following PR testing vs. extinction/reinstatement testing. Radioimmunoassays were conducted at the University of Virginia Center for Research in Reproduction Ligand Assay and Analysis Core.

### Drugs

Cocaine hydrochloride was obtained from the National Institute on Drug Abuse and prepared in sterile saline (7 mg/ml). The mg/kg dose was adjusted for changes in body weight three times a week by adjusting the infusion duration. Tamoxifen and sesame oil (vehicle) were purchased from Sigma-Aldrich (St. Louis, MO, USA).

### Data Analysis

Intake during the ExA component of the study was compared between groups using a linear mixed-effects model with group, session, and their interaction as fixed factors and subject as a random effect. A paired-samples *t*-test was used to compare average intake between sessions 1–2 vs. 3–10. The primary measure for the development of an addiction-like phenotype was an enhanced motivation for cocaine which was determined following ExA self-administration and abstinence and, based on our previous findings in intact males and females (Lynch and Taylor, 2004; Ramôa et al., 2013, 2014; Doyle et al., 2014), was defined as a 15% or greater increase in PR responding during retest as compared to baseline (averaged across the three sessions within each phase). A linear mixed-effects model was also used to examine group differences in PR responding for cocaine (number of infusions obtained during the three baseline and retest sessions) with phase (baseline vs. retest) as an additional fixed factor. *Post hoc* comparisons within each group were made using Bonferroni-corrected paired *t*-tests and within phase *post hoc* comparisons to control (VEH group) were performed using Dunnett’s *t*-tests. In order to control for group differences in PR responding at baseline, data were also examined as percent change from baseline to retest using similar statistical procedures (i.e., a linear mixed-effects model, Dunnett’s *t*-test for *post hoc* between-group comparisons and Bonferroni-corrected one-sample *t*-tests for within-group comparisons).

Similar procedures were used to examine group differences during extinction (active-lever responses during sessions 1–9), with a paired-samples *t*-test used to compare responding during session 1 vs. later sessions (2–9) and univariate analysis of variance (ANOVA) used to examine differences within each session. Since not all rats required greater than 6 sessions to extinguish responding, zero responses were used in the analyses for rats who had already reached the extinction criteria. We also verified that the effects were similar when analyzed during the first six extinction sessions only. Univariate ANOVAs were used to analyze group differences in total responses on the inactive-lever during extinction and reinstatement, with *post hoc* comparisons made using Dunnett’s *t*-test. A Kruskal-Wallis test was used to compare the number of sessions required to meet the extinction criteria (i.e., 6, 7, 8, or 9). A linear mixed-effects model was used to compare the number of active-lever responses during the last extinction session vs. the reinstatement session. Serum levels of estradiol, progesterone, and the ratio of estradiol to progesterone were collapsed across experiments (PR and reinstatement) and analyzed using univariate ANOVAs with *post hoc* comparisons performed using Dunnett’s *t*-tests. Changes in body weight across 10 time-points from arrival to PR/relapse testing following abstinence (i.e., at arrival and during cocaine self-administration training, PR baseline, early, mid, and late ExA self-administration, early, mid, and late abstinence, and at test) were examined using a linear mixed-effects model with Dunnett’s *t*-tests used for *post hoc* comparisons. One-tailed *t*-tests were used for predicted differences in motivation for cocaine, and two-tailed *t*-tests were used for all other comparisons. Statistical analyses were performed using SPSS (V26). Alpha was set at 0.05. Data are presented as the mean ± SEM.

## Results

### Effect of Tamoxifen on ExA Cocaine Self-administration

**Figure 1:**
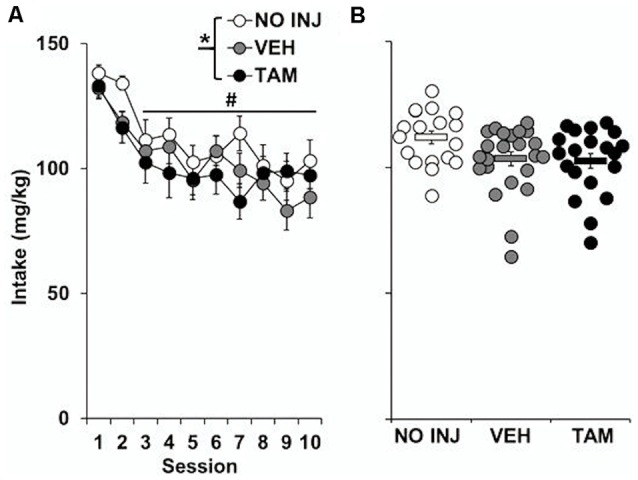
Effect of tamoxifen on ExA cocaine self-administration. **(A)** Mean (±SEM) cocaine intake (mg/kg) for the no-injection (NO INJ; *n* = 18), vehicle (VEH; *n* = 24) and tamoxifen (TAM; *n* = 20) groups for each of the 10 ExA cocaine self-administration sessions. *Significant group effect (*P* = 0.048); ^#^Significant effect of session (1–2 vs. 3–10, *P* < 0.0001) **(B)** Scatterplot of each individual rat’s mean cocaine intake across the 10 days of ExA cocaine self-administration. Solid bars represent the average values within each group (±SEM).

Although a significant effect of group was observed for cocaine intake (*F*_(2,59)_ = 3.190, *P* = 0.048; Figure 1), this effect appears to be due to a non-significant trend for higher intake in the NO INJ group as compared to VEH (*P* = 0.07); no differences were observed between the VEH and TAM groups. All groups self-administered cocaine in a similar pattern (group-by-session, *P* = 0.801), with the highest levels of intake occurring during the first two sessions (session, *F*_(9,531)_ = 11.608, *P* < 0.0001; session 1–2 vs. 3–10, *t* = 12.189, *df* = 61, *P* < 0.0001).

### Effect of Tamoxifen on the Development of an Enhanced Motivation for Cocaine

**Figure 2:**
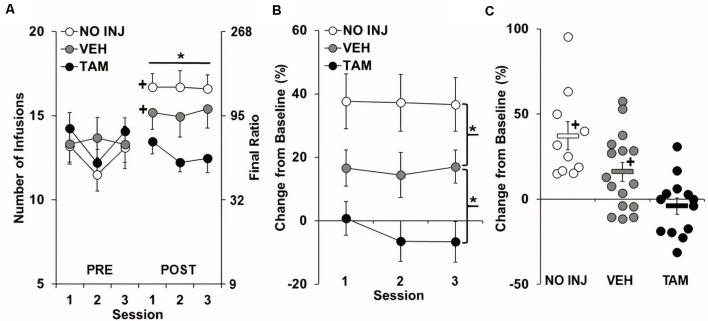
Tamoxifen blocked the development of an enhanced motivation for cocaine. **(A)** Mean (±SEM) number of cocaine infusions obtained under the progressive-ratio (PR) schedule during each of the three sessions at baseline (PRE) and retest following ExA self-administration and abstinence (POST) for the no-injection (NO INJ; *n* = 10), vehicle (VEH; *n* = 16) and tamoxifen (TAM; *n* = 13) groups. *Significant group effect (*P* = 0.022); ^+^Significant increase from baseline within the VEH (*P* = 0.042) and NO INJ groups (*P* < 0.001). **(B)** Percent change (±SEM) in the number of infusions obtained at retest following ExA self-administration relative to baseline (PRE) averaged across the three sessions. *Significant difference between the NO INJ and VEH groups (*P* = 0.044) and the VEH and TAM groups (*P* = 0.019). **(C)** Scatterplot of each individual rats’ data for percent change in the number of infusions from baseline to retest. Solid bars represent the average values within each group (±SEM). ^+^Significant increase from zero within each group (NO INJ, *P* = 0.003; VEH, *P* = 0.036).

As predicted, following ExA self-administration and 14 days of abstinence, PR responding for cocaine increased from baseline for the VEH and NO INJ groups, but not for the TAM group (Figure 2A), with results revealing significant group-by-phase (*F*_(2,180)_ = 22.406, *P* < 0.0001) and phase effects (*F*_(1,180)_ = 35.253, *P* < 0.0001), a trend for a session effect (*F*_(2,180)_ = 2.963, *P* = 0.054), but a non-significant group effect (*P* = 0.452). Subsequent analysis within baseline (Figure 2A, Pre) revealed a trend for a session effect (*F*_(2,72)_ = 3.026, *P* = 0.055), but non-significant group (*P* = 0.785) and group-by-session effects (*P* = 0.176). In contrast, analysis within retest (Figure 2A, Post) revealed a significant group effect (*F*_(2,36)_ = 4.268, *P* = 0.022), with *post hoc* comparisons revealing a trend for higher PR responding in the VEH vs. the TAM group (*P* = 0.054), and no difference between the VEH and NO INJ groups. Subsequent analysis within each group showed that the number of infusions obtained significantly increased from baseline to retest for the NO INJ and VEH groups (*t* = 7.156, *df* = 9, *P* < 0.001; *t* = 2.773, *df* = 15, *P* = 0.042, respectively), but not for the TAM group (*P* = 0.286).

Similar differences in motivation for cocaine were observed in the analysis of percent change from baseline to retest (Figure 2B), with results revealing a significant group effect (*F*_(2,36)_ = 10.088, *P* < 0.0001), but non-significant effects of session (*P* = 0.508) and group-by-session (*P* = 0.784). *Post hoc* comparisons to VEH revealed a significant difference for both the TAM (*P* = 0.019) and NO INJ groups (*P* = 0.044). Analysis within each of the groups revealed that motivation for cocaine increased by 15% or more in all 10 NO INJ rats, 7 of the 16 VEH rats, and 2 of the 13 TAM rats (Figure 2C), with the average percent change found to be significantly increased from baseline (0) for the NO INJ (*t* = 4.496, *df* = 9, *P* = 0.003) and VEH groups (*t* = 2.857, *df* = 15, *P* = 0.036), but not for the TAM group (*P* = 0.405). Together, these findings confirm the development of an addiction-like phenotype in the NO INJ and VEH groups, and show that it is blocked by tamoxifen treatment, and surprisingly, attenuated by vehicle (oil) injections.

### Effect of Tamoxifen on Relapse Vulnerability

**Figure 3:**
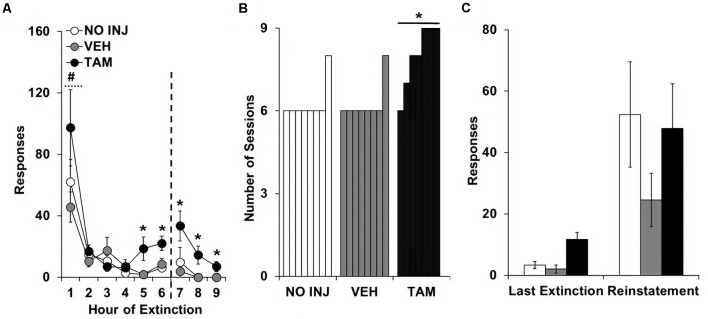
Tamoxifen impaired extinction learning. **(A)** Mean (±SEM) active-lever responses made during the nine extinction sessions for the no-injection (NO INJ; *n* = 8), vehicle (VEH; *n* = 8) and tamoxifen (TAM; *n* = 7) groups. The dotted line indicates that additional extinction sessions were run for rats that did not meet the extinction criterion within the first six sessions (≤15 responses; one of the eight rats in the NO INJ and VEH groups and six of the seven rats in the TAM group). *Significant difference between the VEH and TAM groups (session 5, *P* = 0.017; session 6, *P* = 0.029; session 7, *P* = 0.035; session 8, *P* = 0.007; session 9, *P* = 0.02); ^#^Significant effect of session (1 vs. 2–9, *P* < 0.0001). **(B)** Number of sessions required to extinguish responding (≤15 responses). *Unequal distribution across groups. **(C)** Mean (±SEM) active-lever responses made during the last extinction session and reinstatement test session.

Analysis of responding on the formerly-active lever over the nine extinction sessions revealed significant effects of group (*F*_(2,20)_ = 7.812, *P* = 0.003; Figure 3A), session (*F*_(8,160)_ = 25.759, *P* < 0.0001; session 1 vs. 2–9, *t* = 5.974, *df* = 22, *P* < 0.0001), and group-by-session (*F*_(16,160)_ = 1.743, *P* = 0.044), with *post hoc* comparison to VEH revealing significantly higher responding in the TAM group (*P* = 0.004). Similar effects were also observed in the analysis of the first six extinction sessions, which all rats completed (group, *F*_(2,20)_ = 4.323, *P* = 0.028; session, *F*_(5,100)_ = 28.428, *P* < 0.0001; group-by-session, *F*_(10,100)_ = 1.930, *P* = 0.05). Analysis within each session revealed non-significant effects of group within each of the first four sessions; however, a significant group effect was observed for each of the subsequent sessions (session 5, *F*_(2,20)_ = 5.410, *P* = 0.026; session 6, *F*_(2,20)_ = 5.470, *P* = 0.026; session 7, *F*_(2,20)_ = 3.570, *P* = 0.047; session 8, *F*_(2,20)_ = 6.935, *P* = 0.005; session 9, *F*_(2,20)_ = 5.147, *P* = 0.016). *Post hoc* comparisons to VEH within each session revealed significantly higher responding in the TAM group (session 5, *P* = 0.017; session 6, *P* = 0.029; session 7, *P* = 0.035; session 8, *P* = 0.007; session 9, *P* = 0.02). A significant group difference was also found for inactive-lever responses during extinction (group, *F*_(2,20)_ = 3.521, *P* = 0.049), with *post hoc* comparisons to VEH revealing decreased responding in the NO INJ group (*P* = 0.028; data not shown). The number of sessions required to extinguish responding also differed between groups (*P* = 0.003; Figure 3B), as seven out of eight rats in both the NO INJ and VEH groups extinguished responding within six sessions, while all TAM rats, except one, required more than six sessions. Comparison of responses on the formerly-active lever during the last extinction session vs. the reinstatement session revealed a significant effect of phase (*F*_(1,40)_ = 19.603, *P* < 0.0001; Figure 3C) but non-significant effects of group and group-by-phase indicating that, while responding was reinstated by the cues formerly associated with cocaine, this occurred similarly between the groups. Thus, while tamoxifen impaired the extinction process, it did not affect initial responding during extinction or responding during reinstatement.

### Effect of Tamoxifen on Serum Hormone Levels

**Figure 4:**
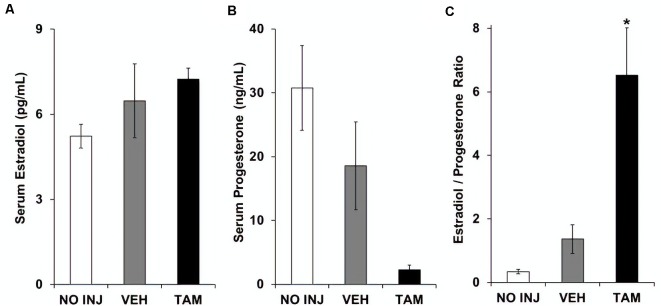
Tamoxifen was associated with an increased ratio of serum estradiol to serum progesterone. **(A)** Mean (±SEM) serum estradiol concentration in the no-injection (NO INJ, *n* = 14), vehicle (VEH, *n* = 12), and tamoxifen (TAM, *n* = 11) groups. **(B)** Mean (±SEM) serum progesterone concentration. Despite a significant overall effect of group (*P* = 0.006), Dunnett-corrected pair-wise comparisons to the VEH group were not significant due to a high level of variability in this group. **(C)** Mean (±SEM) ratio of estradiol to progesterone. *Significant difference between the VEH and TAM groups (*P* < 0.0001).

Each of the groups had similar serum levels of estradiol (group effect, *P* = 0.212; Figure 4A). While a significant group effect was observed for progesterone (*F*_(2,34)_ = 5.948, *P* = 0.006; Figure 4B), *post hoc* comparisons to the VEH group were not significant due to a high level of variability in this group (*P*’s > 0.10). The ratio of estradiol to progesterone differed between groups (*F*_(2,34)_ = 16.102, *P* < 0.0001; Figure 4C), with a higher ratio observed in the TAM group vs. the VEH group (*P* < 0.0001). Thus, while tamoxifen did not significantly affect either estradiol or progesterone, it increased the ratio of estradiol to progesterone.

### Effect of Tamoxifen on Body Weight

**Figure 5:**
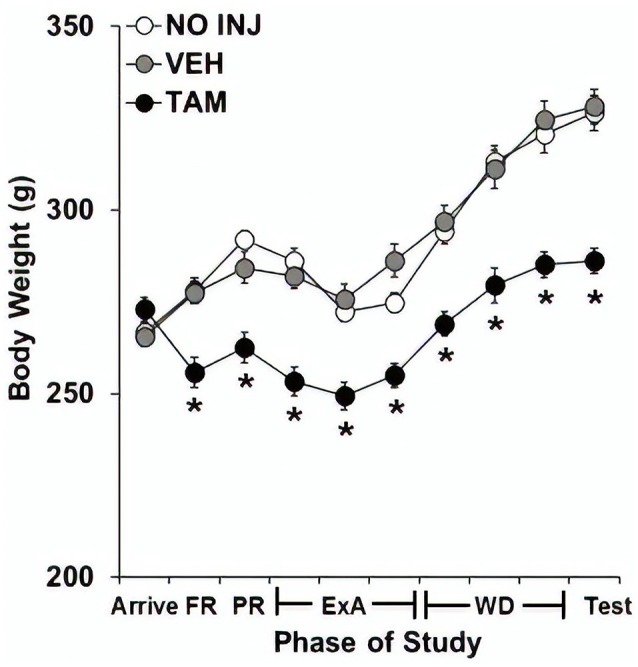
Tamoxifen decreased body weight. Mean (±SEM) body weight across 10 phases of study for the no-injection (NO INJ, *n* = 18), vehicle (VEH, *n* = 24), and tamoxifen (TAM, *n* = 20) groups. *Significant difference between the VEH and TAM groups (*P*’s < 0.0001). FR, fixed ratio; PR, progressive-ratio; ExA, extended-access; WD, withdrawal/abstinence.

Body weights were markedly reduced in the TAM group compared to the NO INJ and VEH groups (Figure 5), as analysis across the 10 phases of the study revealed significant effects of group (*F*_(2,59)_ = 24.195, *P* < 0.0001), phase (*F*_(9,531)_ = 130.605, *P* < 0.0001) and group-by-phase (*F*_(18,531)_ = 8.604, *P* < 0.0001) with *post hoc* comparisons to the VEH group revealing significantly lower weight in the TAM group (*P* < 0.0001). Subsequent analyses within each phase revealed significant effects of group (*P*’s < 0.0001) as well as significantly lower weight in the TAM vs. VEH group at every phase except arrival (*P*’s < 0.0001).

## Discussion

The purpose of this study was to determine the role of estradiol in the development of an addiction-like phenotype in ovary-intact females treated chronically with tamoxifen. As predicted, tamoxifen prevented the increase in motivation for cocaine following ExA self-administration and protracted abstinence, suggesting that ER signaling is critical for the development of this feature of an addiction-like phenotype. Surprisingly, the increase in motivation for cocaine following abstinence was also attenuated in the vehicle group as compared to no-injection controls, suggesting that oil/injections also affected the development of this feature of an addiction-like phenotype. Contrary to our hypothesis, tamoxifen did not decrease vulnerability to relapse as this group responded at similar levels during initial extinction sessions (1–4) and cue-induced reinstatement testing as compared to controls. Tamoxifen did, however, impair extinction learning as this group continued to respond at high levels during later extinction sessions and took longer to extinguish as compared to controls. Taken together, these findings indicate that estradiol is critical for the extinction of drug-associated cues and the development of motivational features of addiction.

As predicted, motivation for cocaine was increased following ExA self-administration and protracted abstinence in the NO INJ and VEH groups (37.2% and 16%, respectively), but not in the TAM group (−4.1%). These findings are consistent with previous work showing that OVX prevents the development of an enhanced motivation for cocaine following ExA self-administration and protracted abstinence, as well as the development of a preference for cocaine over food, while estradiol replacement restores these phenotypes (Kerstetter et al., 2012; Ramôa et al., 2013, 2014). That similar findings were observed here in ovary-intact females as compared to previous work in OVX females indicates that the effects of estradiol are reliable and robust. Taken together, these findings strongly support the hypothesis that estradiol is critical for the development of motivational features of an addiction-like phenotype, and likely underlies the accelerated time-course for the development of addiction in women and an addiction-like phenotype in female laboratory animals (Lynch and Carroll, 2000; Lynch and Taylor, 2004; Center for Substance Abuse Treatment, 2009; Greenfield et al., 2010; Becker and Koob, 2016; Kawa and Robinson, 2019; Nicolas et al., 2019).

While the mechanisms underlying these effects are not yet clear, they likely involve estradiol-dopamine interactions in the reward pathway. Numerous studies have shown that estradiol enhances drug-induced increases in dopaminergic signaling in the ventral tegmental area and striatum (Becker, 1990a,b; Becker and Rudick, 1999; Russo et al., 2003; Song et al., 2019). Results also show that antagonizing ER signaling, either with tamoxifen, ICI 182, 780, or ER knockdown, offsets estradiol-induced increases in striatal dopamine release and decreases the rewarding effects of drugs of abuse (Walf et al., 2007; Satta et al., 2018; Song et al., 2019). There is also evidence indicating that the development of an enhanced motivation for cocaine in females may depend on estradiol’s ability to potentiate dopaminergic signaling during initial exposure. Specifically, Calipari et al. (2017) showed that females conditioned during proestrus/estrus, when levels of estradiol are relatively high, had a heightened behavioral and neurochemical response to cocaine as compared to females conditioned during diestrus. In addition, Johnson et al. (2019) further demonstrated that only cues that had initially acquired their value during estrus led to a subsequent increase in motivation for cocaine when compared to males or females initially trained during diestrus. Thus, it is possible that ER-induced amplification of dopaminergic signaling in the ventral tegmental area and NAc, possibly during initial drug exposure, underlies the accelerated time-course observed in females with estradiol. It is also possible that the effects of estradiol/ER signaling are mediated *via* other signaling pathways. For example, we previously showed that the development of an addiction-like phenotype is accompanied by a shift in the mechanism motivating cocaine self-administration, from NAc dopamine to glutamate (Doyle et al., 2014; Ramôa et al., 2014). We further showed that OVX prevented not only the behavioral phenotype, but also the diminished role for dopaminergic signaling in the NAc (Ramôa et al., 2014). Thus, an alternative, non-mutually exclusive possibility is that estradiol is necessary for the shift from NAc dopamine to glutamate. Future research is necessary to investigate these possibilities. Additionally, since tamoxifen was administered throughout the study, future research is needed to determine which time-points during the development of an addiction-like phenotype that estradiol is critical (i.e., during initial exposure, ExA self-administration, or abstinence). Such studies are also necessary to address the possibility that tamoxifen prevents the expression rather than the development of an addiction-like phenotype.

Interestingly, unlike OVX (Lynch and Taylor, 2005; Ramôa et al., 2013, 2014; Martinez et al., 2016), tamoxifen treatment did not significantly decrease cocaine intake during ExA self-administration, yet both OVX and tamoxifen prevent the subsequent increase in motivation for cocaine (Ramôa et al., 2013, 2014). These discrepant results could indicate a less robust effect of estradiol on intake vs. motivational features of an addiction-like phenotype; however, future research will be necessary to resolve this inconsistency given that the effects reported previously with OVX appear to be robust and reliable for both intake and motivational features of an addiction-like phenotype (Lynch and Taylor, 2005; Ramôa et al., 2013, 2014; Martinez et al., 2016). The effects of tamoxifen treatment in intact females are also likely very different than the effects of OVX and estradiol replacement. The fact that intake did not differ in the current study is nonetheless a strength of the tamoxifen ovary-intact model, considering that reduced intake was a confounding factor in previous studies investigating the role of estradiol in OVX rats.

Contrary to our hypothesis, tamoxifen did not decrease relapse vulnerability as responding during both the initial extinction sessions and reinstatement testing were similar between the groups. These findings are consistent with results in both women and female rats showing that levels of progesterone, but not estradiol, are predictive of cue-induced craving/seeking (Feltenstein and See, 2007; Sinha et al., 2007). However, they are in contrast to results from studies examining drug-primed reinstatement, which show that the reinstatement of drug-seeking is decreased by OVX and restored by estradiol replacement (Larson et al., 2005; Anker et al., 2007; Larson and Carroll, 2007). Thus, while these findings indicate that the role of ER signaling is different for relapse vs. motivational features of addiction, further research is necessary to determine its role under other relapse testing conditions, particularly in response to drug primes.

We also observed a paradoxical increase in extinction responding as a consequence of tamoxifen treatment. This effect appears to be due to impairment of extinction learning as the tamoxifen group continued to respond at high levels, even after responding had extinguished in controls. This interpretation is consistent with recent results in intact females showing that estradiol, through its learning enhancing functions, can be used to facilitate the extinction of cocaine-seeking following cocaine self-administration (Yousuf et al., 2019). Similar findings have also been observed in OVX female rats where estradiol markedly accelerated the extinction of a cocaine-induced place preference leading to extinguished expression in 8 days vs. over a month in vehicle-treated controls (Twining et al., 2013). Indeed, learning and memory varies across the menstrual/estrous cycle (Frick and Berger-Sweeney, 2001; Frye et al., 2007; Paris and Frye, 2008; Pompili et al., 2010; Luine and Frankfurt, 2013; Frick et al., 2015; Kromrey et al., 2015), is impaired by both tamoxifen treatment and OVX (Chen et al., 2002; Rissman et al., 2002; Heikkinen et al., 2004; Sarkaki et al., 2008; Esmaeili et al., 2009; Su et al., 2012; Twining et al., 2013; Lichtenfels et al., 2017; Djiogue et al., 2018), and can be restored in OVX rats by estradiol replacement (Luine et al., 1998; Gibbs, 2000; Frye and Rhodes, 2002; Rhodes and Frye, 2004; Frye et al., 2005; Gresack and Frick, 2006; Jasnow et al., 2006). These learning enhancing effects of estradiol may serve to both heighten vulnerability to addiction by enhancing drug-associated learning, and paradoxically reduce vulnerability by facilitating the extinction of drug-associated learning.

We also observed modest, but surprising, protective effects of vehicle treatment on the development of an enhanced motivation for cocaine as this group showed less of an increase in motivation for cocaine as compared to the non-treated controls. Additionally, while 100% of non-treated controls showed a 15% or more increase in motivation for cocaine following protracted abstinence, only 44% of the vehicle-treated group displayed this phenotype. We selected sesame oil for the vehicle in this study as it is commonly used to dissolve fat-soluble hormones in not only addiction studies (Perrotti et al., 2000; Roth-Deri et al., 2006; Silverman and Koenig, 2007; Russo et al., 2008; Mello et al., 2011; Van Swearingen et al., 2013; Ghazvini et al., 2016; Rauhut and Curran-Rauhut, 2018), but also in general biomedical studies (Dubal et al., 2001; Babaei et al., 2010; Asarian et al., 2012; McClure et al., 2013; Hiroi et al., 2016; González-Garcia et al., 2018; Khariv et al., 2018; Matsumoto et al., 2018). Given its widespread use, we were reluctant to attribute effects in the vehicle group to sesame oil. However, it is a strong possibility given findings from two recent studies with fish oil. Specifically, these studies showed that chronic treatment with fish oil, which like sesame oil is rich in essential polyunsaturated fatty acids (Sowmya et al., 2009), prevented the reinstatement of an amphetamine or morphine-induced CPP and associated molecular changes (Metz et al., 2019; Milanesi et al., 2019). Sesame oil is also rich in linoleic acid, which has been reported to have antagonistic effects on ER signaling (Durgam and Fernandes, 1997; Kenny et al., 2000; Tanmahasamut et al., 2004; Liu and Sidell, 2005). In fact, one study found that while estradiol dissolved in propylene glycol produced a conditioned place preference in OVX female rats, when dissolved in sesame oil, this same dose of estradiol failed to induce a conditioned place preference (Frye and Rhodes, 2006). The other possibility is that effects are due to stress from chronic subcutaneous injections, an alternative that will be addressed in future studies by measuring corticosterone levels. However, this possibility seems less likely considering that the stress associated with chronic injections should enhance, rather than reduce, vulnerability (Goeders and Guerin, 1994; Piazza and Le Moal, 1998). Further research is needed to address the potential mechanism for effects observed following vehicle treatment.

There are two potential confounds in this study. First, we observed significantly diminished weight gain with tamoxifen treatment. This is a seemingly unavoidable confound that occurs as a consequence of chronic estradiol manipulation. Indeed, OVX also dramatically impacts body weight and is thus plagued with the same confound (Roesch, 2006; Wegorzewska et al., 2008). While direct manipulation of ERs in the brain through site-specific infusion would likely minimize effects on body weight (Wade and Heller, 1993; Wade et al., 1993; Sibonga et al., 1998), such techniques also limit translational value. Second, although we did not observe a significant impact of tamoxifen treatment on serum levels of estradiol or progesterone, likely due to the high variability in the vehicle group, tamoxifen treatment did produce markedly higher ratios of estradiol to progesterone, as would be expected given previous findings (Sibonga et al., 1998; Wilson et al., 2003; Messinis, 2006). However, it is important to note that given the complex nature of the positive and negative feedback mechanisms regulating estradiol, progesterone, FSH, and LH release (Kubota et al., 2016), all hormone manipulation models are confounded by unintended effects on hormone levels and hormone-dependent behaviors (i.e., anxiety and depression; Azizi-Malekabadi et al., 2015).

In summary, tamoxifen prevented the development of an enhanced motivation for cocaine following ExA self-administration and abstinence indicating that ER signaling is critical for the development of motivational features of addiction and likely contributes to the accelerated time-course observed in females for the development of addiction. Contrary to our hypothesis, however, tamoxifen did not decrease vulnerability in response to cocaine-associated cues indicating that the role of ER signaling in relapse may differ from its role in motivating cocaine use. However, future research is necessary to examine its role in other forms of relapse (e.g., drug-primed). Tamoxifen also impaired the extinction of cocaine-seeking indicating that ER signaling may be critical for not only establishing and maintaining drug self-administration but also for facilitating new drug-associated learning during extinction training. Future research is needed to determine the mechanisms that underlie estradiol’s differential effects on relapse, extinction learning, and motivational features of addiction.

## Data Availability Statement

The raw data supporting the conclusions of this manuscript will be made available by the authors, without undue reservation, to any qualified researcher.

## Ethics Statement

All animal protocols were reviewed and approved by The University of Virginia Animal Care and Use Committee.

## Author Contributions

AB-S and WL designed the study, performed the statistical analysis, and wrote the manuscript. AB-S and TN collected the data. All authors contributed to manuscript revision, read and approved the submitted version.

## Conflict of Interest

The authors declare that the research was conducted in the absence of any commercial or financial relationships that could be construed as a potential conflict of interest.
